# Assessing nursing mothers’ knowledge, perceptions and uptake of Sulphadoxine Pyrimethamine (IPTp-SP) during pregnancy in the Ho Teaching Hospital of the Volta Region of Ghana

**DOI:** 10.1371/journal.pgph.0000904

**Published:** 2023-02-10

**Authors:** Verner N. Orish, Prince N. Puplampu, Sylvester Y. Lokpo, Precious K. Kwadzokpui, Virtue Fiawokome De-Gaulle, Aleksandra Marinkovic, Stephanie Prakash, Rochelle Annan, Chuku Okorie, Adekunle Sanyaolu

**Affiliations:** 1 Department of Microbiology and Immunology, School of Medicine, University of Health and Allied Sciences, Ho, Volta Region, Ghana; 2 School of Medicine, University of Health and Allied Sciences, Ho, Ghana; 3 Department of Medical Laboratory Sciences, School of Allied Health Sciences, University of Health and Allied Sciences, Ho, Ghana; 4 Laboratory Department, Ho Teaching Hospital, Ho, Volta Region, Ghana; 5 Faculty of Medicine and Biomedical Sciences, University of Yaoundé, Yaoundé, Cameroun; 6 Saint James School of Medicine, The Quarter, Anguilla; 7 School of Medicine, University of Health Sciences Antigua, Antigua; 8 Union County College, Plainfield Campus, Plainfield, New Jersey, United States of America; 9 Federal Ministry of Health, Abuja, Nigeria; The University of Texas Health Science Center at Houston School of Public Health, UNITED STATES

## Abstract

Intermittent preventive therapy during pregnancy with Sulphadoxine Pyrimethamine (IPTp-SP) is one of the potent strategies for preventing malaria in pregnancy (MiP). Factors such as the pregnant woman’s knowledge and very importantly perception or belief about IPTp-SP remains key determinant of IPTp-SP uptake. This study assessed the knowledge and perception of nursing mothers and their uptake of IPTp-SP during pregnancy in the Ho Teaching Hospital. We administered a close-ended questionnaire to 303 nursing mothers and obtained their sociodemographic details as well as information on their knowledge and perception of IPTp-SP utilization. We also reviewed the nursing mothers’ antenatal care (ANC) booklets to ascertain the number of times IPTp-SP was taken during pregnancy. Pearson chi-square was used to determine the association between the sociodemographic variable and the categories of knowledge, perception, and uptake of IPTp-SP. Analysis, was done using SPSS, and the p-value of less than 5% was considered statistically significant. Of the 303 nursing mothers sampled in this study, 265(87.5%) had heard about IPTp-SP of which 138(52.1%) had average knowledge of IPTp-SP. A total of 168(63.4%) had poor perception, and 168(64.6%) had adequate uptake (3–4 doses) of IPTp. Education was significantly associated with perception and uptake, with the majority of women who demonstrated excellent perception were those who had tertiary education (7, 6.35%, p = 0.05), and the majority who demonstrated excellent uptake (5 completed doses) were women who had tertiary education (47, 37.9%, p = 0.01). While knowledge was average, perception of IPTp-SP was poor for the majority of the nursing mothers which might have hampered their uptake of IPTp-SP during pregnancy. It is important that continuous assessment of the individual factors affecting the uptake of IPTp-SP be done regularly to curb the negative influences on the uptake of IPTp-SP.

## Introduction

Malaria in pregnancy remains a significant public health problem resulting in morbidity and mortality for both the mother and fetus [1]. Malaria in pregnancy was reportedly responsible for over 200,000 infant deaths and about 10 thousand maternal deaths yearly, globally [2] for the past two decades. In 2019, about 11 million pregnant women reportedly suffered malaria resulting in over 800,000 children being born with low birth weight [3]. Preventing malaria in pregnancy became a priority of the rollback malaria initiative with a three-prong approach consisting of the use of insecticide-treated bed net (ITN), effective case management, and the use of intermittent preventive treatment using Sulphadoxine-pyrimethamine during pregnancy (IPTp-SP) [1]. Sulphadoxine-pyrimethamine combination became the drug of choice in the Intermittent prevention treatment strategy in controlling malaria in pregnancy [4].

IPTp-SP has been extensively used among pregnant women in malaria-endemic areas, where multiple dozes-up to 5 dozes is given every 4 weeks from the 16 weeks of pregnancy [5]. Although progress has been made in the implementation of the IPTp-SP program, it is also experiencing some challenges that hamper its success, with less than 50% of pregnant women who attend antenatal care (ANC) receiving the recommended doses of IPTp-SP [3]. These challenges include; health system failures such as stock out of Sulphadoxine-pyrimethamine and failure of health care workers in dispensing IPTp-SP to the pregnant women [6]; and individual factors of the pregnant women like refusal to take IPTp-SP which might be affected by poor knowledge and poor perception of the IPTp-SP [7]. These individual factors have contributed significantly to the poor uptake of IPTp-SP and pose a significant threat to the prevention program [7].

In Ghana, malaria causes significant morbidity and mortality among pregnant mothers and their fetuses [8]. In 2014, malaria in pregnancy accounted for about 17.6% of all hospital visits and 13.7% of admission among pregnant women, and 3.4% of maternal deaths [9]. Although there has been a considerable decline, there is still a significant number of infections in pregnant women each year [9]. With the IPTp-SP program still bedeviled by poor health system factors in the country, poor knowledge and poor perception of the IPTp-SP is aggravating the problem [10–12]. It is important to constantly assess the knowledge and perception of these women and how these affect their uptake of IPTp-SP. This study aimed at assessing the uptake of IPTp-SP during pregnancy among nursing mothers as well as their knowledge and perception of IPTp-SP.

## Methods

### Study site and setting

The study was a cross-sectional study conducted in the Maternity Ward and Child Welfare Clinic of the Ho Teaching Hospital (HTH). The Ho teaching hospital is the main referral facility in the Volta Region with a 300+ bed capacity and comprises 5 departments including internal medicine, surgery, obstetrics and gynecology, child health, and public health. It serves patients from Ghana and the neighboring West African Countries. The HTH has about 6000 pregnant women coming for ANC yearly, and about 2000 women for deliveries and post-natal care yearly. The Ho municipality according to the 2010 population and housing census, has a population of 177,281 which is approximately 8.4% of the region’s population. Women constitute about 52.7% of the population and **most** inhabitants of the municipality (90.3%) are literate.

### Study population and eligibility criteria

This study recruited postpartum nursing mothers aged 18–40 years between 2^nd^ of August and 18^th^ of August 2021 who carried their pregnancy to term and delivered not more than 2 weeks before this study. Both mothers who delivered in the hospital and outside the hospital such as home delivery and reported to the hospital for the necessary postnatal screenings and checkups were included in the study. Nursing mothers who delivered with complications like preeclampsia, eclampsia, postpartum haemorrhage and were under intensive care for any other reason were excluded from the study. Healthy nursing mothers who refused to participate and those who refused consent to participate were excluded from the study. The sampling frame for this study was the register of the maternity ward as well as that of the child welfare clinic. These women were recruited from both the maternity ward and child welfare clinic of the hospital.

### Study design

This study is designed as both quantitative and qualitative (mixed) methods. The quantitative method assesses the uptake of IPTp-SP during pregnancy among nursing mothers in the study using current data retrieved from their ANC booklet, while the qualitative method is questionnaire based to assess the nursing mother’s knowledge and perception of IPTp-SP.

### Questionnaire development and validation

A structured closed-ended questionnaire developed de novo for purposes of this study was used for the data collection process (S1 Questionnaire). Prior to the administration of the questionnaire, a thorough face validation exercise was carried out where two independent experts were asked to objectively review and judge the operation of the various constructs used in the questionnaire. The independent reviewers assessed the questionnaire on how easy it was for the respondents to comprehend the questions in the questionnaire as well as the feasibility, readability, style formatting and clarity in language and the tolerability of the number of questions or items the respondents must provide answers to. The results of the face validation confirmed that the prepared questionnaire format and the presentation of items contained in it were pertinent for consideration as measuring instrument in this study and that the items framed were rational, clear and explicit to be understood by the respondents hence the expert’s opinion were deemed enough for the questionnaire to be used for this study.

### Data collection process

The data collection process for this study was preceded by an initial visit to the ANC of the HTH to inform the ANC nurse in-charge about the project and to solicit for their support. The aim and objectives of the study was explained to the ANC in-charge to bring her up to speed and to also seek for her opinion on other variable worth assessing to ensure the relevance of the study outcome. The data collection took place at the ANC of the hospital. The questionnaire was administered to the nursing mothers by the researchers to obtain their sociodemographic details and their knowledge and perception of IPTp-SP uptake. Additionally, the nursing mother’s overall uptake of IPTp-SP during pregnancy was assessed using current data retrieved from their ANC booklet. A thorough explanation of the details of the questionnaire was ensured to obtain accurate information from the nursing mothers. The questionnaire was administered in the language best understood by the nursing mother such as English, Ewe, and Twi.

### Data management and security

Data collected were imputed into a Microsoft excel 2016 spreadsheet for data verification and cleaning and subsequently exported into the Statistical Package for the Social Sciences (SPSS) version 22 for analysis. For purposes of data security, data obtained was kept in encrypted folder accessible only by researchers.

### Variables

This study included the following variables that have been associated with malaria prevalence and IPTp-SP uptake in the literature. Outcome variables include the level of Knowledge about IPTp-SP defined as average, below average, and above-average; perception about IPTp-SP defined as poor perception, good perception, and excellent perception and level of IPTp-SP uptake defined as poor uptake, adequate uptake, and excellent uptake. Explanatory variables included maternal age, education level, occupation, marital status, religion, residence, gravidity, and parity. Knowledge and Perception about IPTp-SP uptake were also tested against the outcome variable level of IPTp-SP uptake to determine their effect on IPTp-SP uptake among the nursing mothers.

### Sample size

At a confidence level of 95% with a critical value of (z), an estimated IPTp-SP uptake prevalence (p) of 82.1% (12) and an allowable margin of error (e) of 5%, a minimum sample size (n) of 226 was determined using the Cochran formula, n = $\frac{z^{2}p(p-1)}{e^{2}}$.

### Data analysis

Frequency distributions, as well as proportions, were computed for, the sociodemographic characteristics, responses to questions related to the knowledge and perception of the nursing mothers, and the doses of IPTp-SP taken during pregnancy. Correct responses to questions on knowledge and perception were scored 1 and incorrect responses scored 0. The level of knowledge (maximum score = 6) was categorized into average (score = 3), below average (score <3), and above-average (score>3). Nursing mother’s perception (maximum score = 10) was categorized into poor perception (score ≤4), good perception (score = 5–7), and excellent perception (score ≥8) while uptake of IPTp-SP was categorized into poor uptake (less than 3 doses), adequate uptake (3–4 doses) and excellent uptake (5 completed doses). Pearson chi-square was used to determine the association between the sociodemographic variable and the categories of knowledge, perception, and uptake of IPTp-SP. Results were presented as proportions with 95% confidence intervals and findings with a p-value less than or equal to 5% were considered statistically significant.

### Ethical considerations

Ethical approval for this study was obtained from the University of Health and Allied Sciences Research Ethics Committee (UHAS-REC) with ethical clearance certificate number UHAS-REC A. 12 [184] 20–21. In addition, permission was obtained from the authorities of HTH for this study to be carried out. Written **i**nformed consent of the participants was sought before they participated in the study. The participants were assured that no invasive procedure that may cause pain to them would take place as part of the study. To ensure confidentiality, no personal identifiable information such as participant’s name or telephone number was collected. No financial or material benefits was given to the participants in this study. Participation was voluntary and the participants were given the opportunity to truncate their participation in the study at any point of the data collection process if they felt uncomfortable about any of the questions asked.

## Results

This study recruited 303 nursing mothers. The majority were aged 26–35 years (56.1%) and married (78.2%). Similarly, most of the nursing mothers were self-employed (68.3%) and belonged to the Christian religion (88.8%). At the time of the study, the preponderance of the pregnant women had a history of anemia (Hb<11 g/dL) based on their recent HB checked while a little above average (53.5%) delivered babies who weighed between 3.0–3.5kg (Table 1).

**Table 1:** Sociodemographic and clinical characteristics of the study participants.

Variables	Frequency	Percent
Total	303	100.0
**Maternal age**		
≥36	47	15.5
26–35	170	56.1
≤25	86	28.4
**Residence**		
Inside-Ho	188	62.0
Outside-Ho	115	38.0
**Married status**		
Single	19	6.3
Married	237	78.2
Cohabiting	47	15.5
**Education**		
No Education	3	1.0
Primary	15	5.0
JHS	46	15.2
SHS	105	34.6
Tertiary	134	44.2
**Occupation**		
Government sector	74	24.4
Self employed	207	68.3
Unemployed	22	7.3
**Religion**		
Christian	269	88.8
Non-Christian	34	11.2
**Gravidity**		
One–two	156	51.5
Three	77	25.4
Four and more	70	23.1
**Parity**		
One–two	164	54.1
Three	82	27.1
Four and more	57	18.8
**Last maternal hemoglobin checked**		
Normal (≥11.0)	119	39.3
Anemic (<11.0)	184	60.7
**Birthweight of baby**		
<3.0	44	14.5
3.0–3.5	162	53.5
>3.5	97	32.0

In this study, 38 (12.5%) of the nursing mothers had not heard about the IPTp-SP and thus were not eligible for the subsequent questions. Of the 87.5% (265/303) who responded knowing the use of IPTp-SP, only a little above average (51.3%) correctly pointed out malaria prevention as the main purpose of IPTp-SP. Nonetheless, 86.8% and 47.9% indicated that IPTp-SP should be taken up to 5 five times and at least three times respectively during the pregnancy. Most of the pregnant women (240, 98%) however did not know the recommended schedule of IPTp-SP uptake in Ghana. Overall, we found that a little above half of the nursing mothers, 52.1% (138/265) had average knowledge of IPTp-SP utilization (Table 2).

**Table 2:** Knowledge of nursing mothers on IPTp-SP utilization.

Knowledge on IPTp-SP	Responses	No.	%
Heard of IPTp-SP Drug (n = 303)	Yes	265	87.5
	No	38	12.5
Do you know what IPTp-SP is used for? (n = 303)	Yes	265	87.5
	No	38	12.5
Why are IPTp-SP taken during pregnancy? (n = 265)	Ensure weight Gain for the baby	21	7.9
	Make baby strong and healthy	108	40.8
	Prevent malaria during pregnancy	136	51.3
Number of times IPTp-SP should be taken during pregnancy (n = 266)	Don’t Know	16	6.0
	Once	0	0.0
	Twice	0	0.0
	Three times	1	0.4
	Four times	18	6.8
	Five times	231	86.8
Minimum times IPTp-SP should be taken (n = 265)	Don’t Know	65	24.5
	Once	0	0.0
	Twice	0	0.0
	Three times	63	23.8
	Four times	127	47.9
	Five times	10	3.8
Stage of pregnancy to start taking IPTp-SP (n = 265)	Don’t know	1	0.4
	One month after conception	1	0.4
	Before quickening	1	0.4
	After quickening	115	43.4
	At 16 weeks	147	55.5
	After 36 weeks	0	0.0
Is there a schedule of intake of IPTp-SP? (n = 265)	No	20	7.5
	Yes	245	92.5
The recommended schedule of IPTp-SP intake (n = 245)	Don’t Know	240	98.0
	Every two weeks	1	0.4
	Monthly	4	1.6
Knowledge score (n = 265)	Below average	118	44.5
	Average	138	52.1
	Above average	9	3.4

Assessment of the perception of the nursing mothers on the benefits of IPTp-SP in pregnancy revealed that the preponderance of the participants (99.2%) had the perception that IPTp-SP was effective in preventing malaria. However, the majority of the participants did not believe in the effectiveness of IPTp-SP in the prevention of anemia in pregnancy (90.2%) including reducing malaria-associated maternal death (65.6%), improving fetal weight (92.2%), and prevention of intrauterine death (92.8%). Overall analysis showed that most of the nursing mothers, 63.4% (168/265) had poor perceptions about IPTp-SP utilization (Table 3).

**Table 3:** Perception of the benefits of IPTp-SP.

Perception of the benefits of IPTp-SP	Response	n	%
Prevent Anemia in pregnancy (n = 244)	Yes	24	9.8
	No	220	90.2
Reduce Maternal Death (n = 244)	Yes	84	34.4
	No	160	65.6
Prevents Infant death (n = 244)	Yes	156	63.9
	No	88	36.1
Prevent Malaria during pregnancy (n = 244)	Yes	242	99.2
	No	2	0.8
Improves Fetal weight (n = 244)	Yes	19	7.8
	No	225	92.2
Prevents Spontaneous Abortion (n = 256)	Yes	238	89.8
	No	27	10.2
Prevents Intrauterine Death (n = 256)	Yes	19	7.2
	No	246	92.8
Prevents Low Birth Weight (n = 265)	Yes	129	48.7
	No	136	51.3
Prevents Intra-Uterine Growth Restriction (n = 265)	Yes	9	3.4
	No	256	96.6
Prevents Prematurity (n = 265)	Yes	123	46.4
	No	142	53.6
Perception scores (n = 265)	Poor perception	168	63.4
	Good perception	89	33.6
	Excellent perception	8	3.0

**Fig 1:**
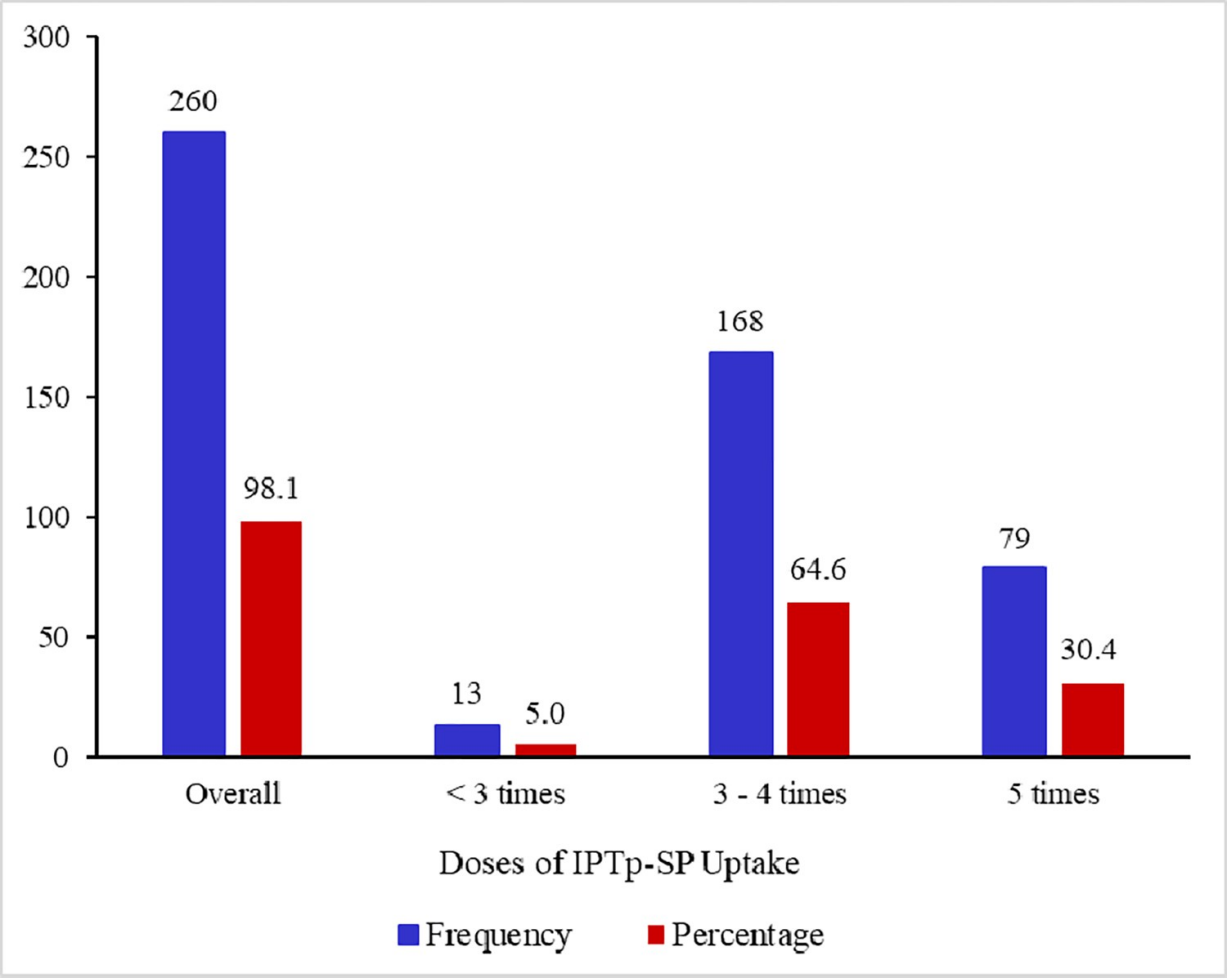
Intake of IPTp-SP of the nursing mothers during pregnancy.

Of the 265 (87.5%) eligible nursing mothers assessed, 259 (97.7%) responded to taking IPTp-SP during pregnancy with 79 (30.4%) of them indicating that they had taken it more than once. Nonetheless, when we reviewed the ANC booklet of 260 nursing mothers who had their booklets available, it was revealed that 5.0% (13/260) of them took less than 3 IPTp-SPs during pregnancy (poor uptake) whereas 64.6% (168/260) and 30.3% (79/260) took 3–4 IPTp-SP (good uptake) and 5 IPTp-SP (excellent uptake) respectively during pregnancy (Fig 1).

Generally, an average level of knowledge was exhibited by most of the nursing mothers studied. We found that average knowledge of IPTp-SP was exhibited by the majority of nursing mothers aged 36 years and above (62.79%), who worked in the government sector (52.71%) and married (54.29%). On the other hand, the poor perception was generally revealed by most nursing mothers towards IPTp-SP. While education did not have any significant association with knowledge of IPTp-SP, education has a significant association with perception with nursing mothers with excellent perception consisting more of women with tertiary education (8, 6.35%, p = 0.05) (Table 4).

**Table 4:** Characteristics of nursing mothers stratified by the level of knowledge and perception.

Variables	Level of knowledge on IPTp-SP uptake				Perception of IPTp-SP uptake			
	Below average	Average	Above average	P-value	Poor	Good	Excellent	P-value
Maternal age								
≤25 years	34[50.75]	30[44.78]	3[4.48]		45[70.31]	18[28.13]	1[1.56]	
26–35 years	69[44.52]	81[52.26]	5[3.23]	0.477	95[62.91]	53[35.10]	3[1.99]	0.149
≥36 years	15[34.88]	27[62.79]	1[2.33]		28[56.00]	18[36.00]	4[8.00]	
Occupation								
Government worker	56[43.41]	68[52.71]	5[3.88]		76[58.46]	47[36.15]	7[5.38]	
Self employed	52[44.83]	61[52.59]	3[2.59]	0.892	78[67.24]	37[31.9]	1[0.86]	0.164
Unemployed	11[52.38]	9[42.86]	1[4.76]		14[73.68]	5[26.32]	0[0.00]	
Location								
In Ho	76[46.91]	80[49.38]	6[3.70]	0.541	104[65.41]	52[32.70]	3[1.89]	0.362
Outside Ho	42[40.78]	58[56.31]	3[2.91]		64[60.38]	37[34.91]	5[4.72]	
Marital status								
Married	88[41.9]	114[54.29]	8[3.81]		125[60.10]	77[37.02]	6[2.88]	
Single	11[50.00]	10[45.45]	1[4.55]	0.404	15[68.18]	6[27.27]	1[4.55]	0.203
Cohabiting	19[57.58]	14[42.42]	0[0.00]		28[80.00]	6[17.14]	1[2.86]	
Religion								
Christian	103[44.59]	121[52.38]	7[3.03]	0.690	144[62.88]	78[34.06]	7[3.06]	0.575
Non-Christian	15[44.12]	17[50.00]	2[5.88]		24[66.67]	11[30.56]	1[2.78]	
Educational Level								
No Education	3[100]	0[0]	0[0.0]	0.1**00**	2[66.67]	1[33.33]	0[0.0]	0.05
Primary	10[66.67]	5[33.33]	0[0.0]		14[93.33]	1[6.67]	0[0.0]	
JHS	15[38.46]	23[58.98]	1[2.56]		25[64.11]	13[33.33]	1[2.56]	
SHS	40[48.78]	39[47.56]	3[3.66]		59[71.95]	23[28.05]	0[0.0]	
Tertiary	50[39.68]	71[56.35]	5[3.97]		68[53.97]	50[39.68]	8[6.35]	

In this study, higher proportions of nursing mothers studied showed an adequate IPTp-SP uptake with over 60% of them with various demographics showing adequate intake of IPTp-SP during gestation. The study showed that higher proportions of single nursing mothers (71.43%) had adequate intake of IPTp-SP than did their married counterparts (63.38%) (Table 5). Nursing mothers, aged 26–35 years (31.17%), married (31.92%), exhibiting good perception (31.82%), and those with average knowledge (32.09%) towards IPTp-SP, had an excellent level of IPTp-SP uptake in this study. The nursing mother’s occupation was significantly associated with the level of IPTp-SP uptake in this study. The educational level of the participants was also observed to have a significant association with nursing mothers with tertiary education having the highest percentage of excellent intake (47, 37.90%; p = 0.01) (Table 5).

**Table 5:** Characteristics of nursing mothers stratified by level of IPTp-SP uptake.

Variables		Level of IPTp-SP uptake			
	Total	Poor	Adequate	Excellent	P-value
Age of Mothers					
<25 years	62	6[9.68]	38[61.29]	18[29.03]	
26–35 years	154	6[3.90]	100[64.94]	48[31.17]	0.404
≥36 years	44	1[2.27]	30[68.18]	13[29.55]	
Occupation					
Government worker	130	3[2.31]	80[61.54]	47[36.15]	
Self employed	112	7[6.25]	77[68.75]	28[25.00]	**0.032**
Unemployed	18	3[16.67]	11[61.11]	4[22.22]	
Location					
In Ho	158	8[5.06]	99[62.66]	51[32.28]	0.699
Outside Ho	102	5[4.90]	69[67.65]	28[27.45]	
Marital status					
Married	213	10[4.69]	135[63.38]	68[31.92]	
Single	14	1[7.14]	10[71.43]	3[21.43]	0.832
Cohabiting	33	2[6.06]	23[69.70]	8[24.24]	
Religion					
Christian	234	10[4.27]	152[64.96]	72[30.77]	0.269
Non-Christian	26	3[11.54]	16[61.54]	7[26.92]	
Educational Level					
No Education	3	1[33.33]	0[0.0]	2[66.67]	**0.010**
Primary	14	1[7.15]	10[71.42]	3[21.43]	
JHS	36	3[8.33]	25[69.44]	8[22.23]	
SHS	83	6[7.23]	58[69.88]	19[22.89]	
Tertiary	124	2[1.62]	75[60.48]	47[37.90]	
Perception about IPTp-SP					
Poor perception	165	11[6.67]	106[64.24]	48[29.09]	
Good perception	88	2[2.27]	58[65.91]	28[31.82]	**<0.001**
Excellent perception	7	0[0.00]	4[57.14]	3[42.86]	
Knowledge on IPTp-SP					
Below average	116	8[6.90]	76[65.52]	32[27.59]	
Average	134	4[2.99]	87[64.93]	43[32.09]	0.478
Above average	10	1[10.00]	5[50.00]	4[40.00]	

## Discussion

The individual factors of pregnant women in endemic areas concerning their knowledge and perception of malaria and its prevention contribute significantly to their acceptance of the IPTp-SP program and their intake of the intervention product [7, 13, 14]. In this study, about 87% of the nursing mothers had heard of IPTp-SP which is much higher than some other studies that reported below 70% awareness of IPTp-SP [13, 14]. The majority (52%) of the nursing mothers had fairly good or average knowledge of IPTp-SP with the majority (51%) of the women answering correctly that IPTp-SP is taken to prevent malaria in pregnancy. This finding (average knowledge of IPTp-SP) is much lower in a study done in another part of the Volta Region where about 85% of pregnant women had average knowledge of IPTp [12]. Knowledge of the number of times and the interval in taking IPTp-SP was also a problem for most of the women in this study and this seems to be a pervasive problem for many pregnant women in malaria-endemic areas [13, 15–17].

The poor perception was a worrying finding in this study as 63% of the women had a poor perception of IPTp-SP. Many of the women did not believe that IPTp-SP can prevent anaemia (90%), improve baby weight at birth (92%), and some did not believe that IPTp-SP can prevent maternal deaths (65%). Questions asked on perception in this study were mainly to probe if the women believe in the effectiveness of the IPTp-SP. This is important because lack of trust or belief might be the reason why some women refuse the IPTp-SP without any reason, despite no reported allergies or side effects [17]. However, unlike this study, other studies show that many pregnant women believe that IPTp-SP is very beneficial for them and their unborn babies [12, 14, 15, 17].

The majority (64%) of the nursing mothers had adequate uptake (3 to 4 doses) of IPTp-SP during their pregnancy with 30% of them noted to have taken the complete 5 doses. A minimum of 3 doses is considered satisfactory, being a recommended dosage by the WHO(5). Despite many studies showing that most pregnant women take the minimum 3 doses of IPTp-SP [12, 15, 18], surveys from endemic countries such as Ghana showed that only about 38% (or less) of pregnant women who attend ANC fall into this category of 3 minimum doses [3, 9]. In the Volta Region, a 2015 survey report showed that less than 35% of pregnant women received 3 or more doses of IPTp-SP [19]. An obvious improvement in health systems and general acceptance of IPTp-SP by pregnant women might explain the higher percentage of uptake of 3 or more doses in this study and a similar study carried out in the Keta District of the Volta Region in 2019 [12]. Ghana, in 2014, adopted the 5-dose policy of IPTp-SP [20], in line with the WHO recommendation that IPTp-SP should be administered at each ANC visit from the second trimester, encouraging more doses of IPTp-SP to be administered at **ANC** to the pregnant women [5]. This recommendation of the WHO is supported by evidence from studies showing that 3 or more doses of IPTp-SP result in higher birth weight and reduced risk of low birth weight compared to 2 doses [5, 21]. In practice, it seems that taking 5 doses of IPTp-SP during pregnancy is a difficulty as many studies have shown many pregnant women fall short of this target [12, 17, 22–24]. This study reported a 30% uptake of 5 IPTp-SP, which is higher than the findings of the studies conducted in Keta (17%) [12], Accra (14%) [23], and Sunyani (9%) [22]. Several challenges might be responsible for this, ranging from pregnant women not registering for ANC on time to stock out of medicines and healthcare workers not adhering to protocols [12, 17, 23].

Taken together, the intake of IPTp-SP during pregnancy in this study (64% take 3 to 4 doses), is somewhat satisfactory considering that most of the women had a poor perception of IPTp-SP during pregnancy. Directly observed therapy (DOT), a highly recommended practice in the administration of IPTp-SP [5, 6], might explain the reason why the majority of the women had 3 or more doses, despite having poor perception. DOT is known to increase the likelihood of pregnant women receiving 3 or more doses [25, 26]. Although DOT does not involve the coercion of pregnant women to take IPTp-SP- as they have the right to refuse- most pregnant women will take IPTp-SP when asked or agree after gentle coaxing, right in the hospital if clean potable water is provided [6, 13, 17].

Education in this study was significantly associated with the perception and intake of IPTp-SP. Nursing mothers with tertiary education proportionally had the highest percentage with excellent perception. Education can improve the knowledge and belief of pregnant women in IPTP, but sometimes educated women have been reported to resist taking IPTp-SP despite much coaxing [17, 27]. Most often, education has been positively linked to increased uptake of IPTp-SP [12, 26, 27], as seen in this study where women with tertiary education were the highest proportion of women who took 3 or more doses of IPTp-SP.

This study has some limitations. Although this study had a qualitative study design section, a more detailed Focus Group Discussion (FGD) would have allowed the women to express themselves further on their knowledge and perception of IPTp-SP. However, despite this limitation, this study highlighted important findings relevant to understanding the individual factors that hamper IPTp-SP uptake by pregnant women.

## Conclusion and recommendation

Most of the pregnant women in this study have heard of IPTp-SP and have average knowledge of IPTp-SP as most of them know that it is given to prevent malaria during pregnancy. The majority of the pregnant women had taken 3 or more doses of IPTp-SP despite many of them having a poor perception of IPTp-SP. Education was associated with a higher intake of IPTp-SP since nursing mothers with tertiary education took 3 or more doses of IPTp-SP during pregnancy. We therefore recommend education to be intensified for the pregnant women on the importance of IPTp-SP both on maternal health and fetal/newborn’s health especially in malaria-endemic regions such as Ghana. Meanwhile, more qualitative studies are needed to fully understand the individual factors that influence the intake of IPTp-SP to enhance policy decisions for the prevention of malaria associated pregnancy.

## Supporting information

S1 Questionnaire Study questionnaire. (DOCX)
