# Supplementary material for: Assessing nursing mothers’ knowledge, perceptions and uptake of Sulphadoxine Pyrimethamine (IPTp-SP) during pregnancy in the Ho Teaching Hospital of the Volta Region of Ghana
Source: PLOS Glob Public Health. 2023 Feb 10;3(2):e0000904. doi: 10.1371/journal.pgph.0000904 (PMC10021858; doi:10.1371/journal.pgph.0000904)
Supplement: S1 Questionnaire — (DOCX) [file pgph.0000904.s001.docx]

**Questionnaire**

**Assessing Nursing Mothers’ Knowledge, Perceptions and Uptake of Sulphadoxine Pyrimethamine (IPTp-SP) During Pregnancy in the Ho teaching Hospital of the Volta Region of Ghana**

| I have given my consent to take part in this study | | | YES |  | NO |  |
| --- | --- | --- | --- | --- | --- | --- |
| Code: ……………………… | | | | Date_____/_____/ 2021 | | |
| 1. General demographic information of respondents | | |  |  | | |
| S/N | Parameters/Questions |  |  | Responds | | |
|  |  | Please tick |  | appropriately as applied to you | | |
| 1 | Maternal age | ………………………………... | | | | |
| 2 | Residence | Inside-Ho |  | Outside-Ho |  | |
| 3 | Marital status | Single |  | Married |  | |
|  |  | Cohabiting |  |  | | |
| 4 | Education | No education |  | Primary |  | |
|  |  | JHS |  | SHS |  | |
|  |  | Tertiary |  |  | | |
| 5 | Occupation | Government sector |  | Self employed |  | |
|  |  | Unemployed |  |  | | |
| 6 | Religion | Christian |  | Non-Christian |  | |
| 7 | Gravidity | One-two |  | Three |  | |
|  |  | Four and more |  |  | | |
| 8 | Parity | One-two |  | Three |  | |
|  |  | Four and more |  |  | | |
| 9 | Last maternal hemoglobin checked | Normal (≥11.0) |  | Anemic (<11.0) |  | |
| 10 | Birthweight of baby | <3.0 |  | 3.0-3.5 |  | |
|  |  | >3.5 |  |  | | |
| 11 | Doses of IPTp-SP Uptake | < 3 times |  | 3-4 times |  | |
|  |  | 5 times |  |  | | |

| 1. **Knowledge on IPTp-SP** | | **Responses** | |
| --- | --- | --- | --- |
| 12 | Heard of IPTp-SP Drug | Yes |  |
|  |  | No |  |
| 13 | Do you know what IPTp-SP is used for? | Yes |  |
|  |  | No |  |
| 14 | Why are IPTp-SP taken during pregnancy? | Ensure weight Gain for the baby |  |
|  |  | Make baby strong and healthy |  |
|  |  | Prevent malaria during pregnancy |  |
| 15 | Number of times IPTp-SP should be taken during pregnancy | Don’t Know |  |
|  |  | Once |  |
|  |  | Twice |  |
|  |  | Three times |  |
|  |  | Four times |  |
|  |  | Five times |  |
| 16 | Minimum times IPTp-SP should be taken | Don’t Know |  |
|  |  | Once |  |
|  |  | Twice |  |
|  |  | Three times |  |
|  |  | Four times |  |
|  |  | Five times |  |
| 17 | Stage of pregnancy to start taking IPTp-SP | Don’t know |  |
|  |  | One month after conception |  |
|  |  | Before quickening |  |
|  |  | After quickening |  |
|  |  | At 16 weeks |  |
|  |  | After 36 weeks |  |
| 18 | Is there a schedule of intake of IPTp-SP? | No |  |
|  |  | Yes |  |
| 19 | The recommended schedule of IPTp-SP intake | Don’t Know |  |
|  |  | Every two weeks |  |
|  |  | Monthly |  |

| 1. **Perception of the benefits of IPTp-SP** | | | | | |
| --- | --- | --- | --- | --- | --- |
| 20 | Prevent Anemia in pregnancy | Yes |  | No |  |
| 21 | Reduce Maternal Death | Yes |  | No |  |
| 22 | Prevents Infant death | Yes |  | No |  |
| 23 | Prevent Malaria during pregnancy | Yes |  | No |  |
| 24 | Improves Fetal weight | Yes |  | No |  |
| 25 | Prevents Spontaneous Abortion | Yes |  | No |  |
| 26 | Prevents Intrauterine Death | Yes |  | No |  |
| 27 | Prevents Low Birth Weight | Yes |  | No |  |
| 28 | Prevents Intra-Uterine Growth Restriction | Yes |  | No |  |
| 29 | Prevents Prematurity | Yes |  | No |  |
| 30 | Perception scores | Yes |  | No |  |
